# Opioid dispensing prior to opioid toxicity hospitalizations and emergency department visits in Canada, 2018–2022

**DOI:** 10.1371/journal.pone.0339643

**Published:** 2026-01-12

**Authors:** Bisola Hamzat, Grace Cheung, Dean T. Eurich, Jean-Luc Kaboré, Louis de Léséleuc, Zhaoyu Liu, Daniel McCormack, Houssem Missaoui, Jason R. Randall, Dana Shearer, David Stock, Tara Gomes

**Affiliations:** 1 Li Ka Shing Knowledge Institute, St. Michael’s Hospital, Toronto, Ontario, Canada; 2 Canadian Institute for Health Information, Toronto, Ontario,; 3 School of Public Health, University of Alberta, Edmonton, Alberta, Canada; 4 Institut National d’Excellence en Santé et en Services Sociaux, Québec, Canada; 5 Canada’s Drug Agency, Ottawa, Ontario, Canada; 6 Centre for Health Informatics, University of Calgary, Calgary, Alberta, Canada; 7 ICES, Toronto, Ontario, Canada; 8 Faculty of Medicine & Dentistry, University of Alberta, Edmonton, Alberta, Canada; 9 Leslie Dan Faculty of Pharmacy, University of Toronto, Toronto, Ontario, Canada; 10 Institute of Health Policy, Management and Evaluation, University of Toronto, Toronto, Ontario, Canada; Government of Nova Scotia, CANADA

## Abstract

**Background:**

Both pharmaceutical and non-pharmaceutical opioids contribute to overdoses and other drug-related harms in Canada; however, the role of these sources of opioids have been dynamic over the past two decades and vary geographically. Therefore, we sought to examine trends in the prevalence of prescription opioid exposure at the time of opioid toxicity hospitalizations and emergency department (ED) visits across Canada over the past 5 years.

**Methods:**

We conducted a population-based repeated cross-sectional study of yearly opioid toxicities treated in an inpatient hospital setting in 6 Canadian provinces―British Columbia, Alberta, Saskatchewan, Manitoba, Ontario, and Quebec―between January 2018 and December 2022. We linked hospitalization records with community pharmacy dispensing data to determine active exposure to prescribed opioids at the time of opioid toxicity, annually and stratified by patient characteristics and type of opioid dispensed. In a secondary analysis, we replicated these methods among opioid toxicities treated in EDs in 5 provinces where data were available.

**Results:**

We identified 23,876 opioid toxicity hospital admissions over the study period. Active opioid exposure at the time of hospital admission declined across provinces over time, apart from British Columbia (range, 20.0% in British Columbia to 47.5% in Quebec in 2018; 19.7% in British Columbia to 36.5% in Quebec in 2022). Generally, people ≥65 and females were proportionally more likely to have an active opioid exposure. When stratified by opioid type, we observed a shift towards active exposure to opioid agonist treatment (OAT), while active exposure to opioids for pain declined over time. Active opioid exposure was less common at the time of opioid toxicity ED visit but was more stable over time (range, 11.0% in British Columbia to 28.5% in Quebec in 2018; 14.9% in Alberta to 26.0% in Quebec in 2022), with only small declines in most provinces (Alberta, Ontario, and Quebec), and an increase in British Columbia (from 11.0% in 2018 to 16.8% in 2022), which was driven by a large increase in active OAT exposure.

**Conclusions:**

Our findings highlight the increasing role of the potent unregulated drug supply in opioid toxicities across Canada, as well as important differences in the role of prescription opioids in harms across demographic groups, geography, and opioid indication.

## Introduction

The drug toxicity crisis is an ongoing public health concern in Canada and the United States, with opioid toxicity harms worsening significantly since 2016 [1,2]. Between January 2016 and June 2024, over 49,000 apparent opioid-related toxicity deaths, 45,000 opioid toxicity hospitalizations and 180,000 opioid toxicity emergency department (ED) visits were reported across Canada, underscoring the scale of the crisis [1]. However, the drug toxicity crisis has evolved over the past decade. While harms were originally attributed to overprescribing and misuse of opioids for pain, the emergence of highly potent synthetic opioids in the unregulated drug supply resulted in a shift in characterization of toxicity harms. In 2023, over 83% of accidental opioid toxicity deaths were attributed to fentanyl in Canada, a 46% increase from 2016, which points to the fentanyl-dominated unregulated drug supply as the primary driver of harms [1]. In line with this, national data also suggests a declining prevalence of pharmaceutical opioid involvement in opioid toxicity deaths over time, with less than 10% of these deaths involving pharmaceutical opioids only (vs. 82% involving non-pharmaceutical opioids only) in 2023 [1]. However, the rate of opioid toxicities, and the role of pharmaceutical and non-pharmaceutical opioids in these harms varies across Canada [1,3]. Underlying provincial differences in socio-economic conditions [4], potency of the unregulated drug supply [5], pharmaceutical opioid prescribing [6–8] and policy structures [9,10,11] shape the opioid environment across jurisdictions, influencing patterns of opioid exposure, access and related harms. For example, while the dominance of fentanyl in the unregulated drug supply has long been tied to high rates of opioid-related deaths in British Columbia [5], evidence points to more recent proliferations of opioid-related harms attributed to fentanyl in Alberta, Manitoba, and Saskatchewan since 2019 [1,3]. Similarly, lower rates of opioid-related toxicity harms in Quebec have been tied to its unregulated drug supply being less dominated by fentanyl [1,5]. Taken together, the complexity of opioid environments across Canadian jurisdictions underscores the importance of assessing regional variations in the extent to which prescribed opioids contribute to opioid-related harms.

Despite evidence of a changing role of pharmaceutical opioids in opioid toxicity deaths, there is a lack of timely data on patterns of opioid exposure before opioid-related toxicities more generally and how these patterns have varied over time, across demographic groups and geography. Specifically, acute care visits related to opioid toxicities occur at a much higher frequency than fatalities in Canada―with opioid toxicity ED visits outnumbering deaths by more than 3-fold in 2023 [1]― highlighting the need for a broader examination of contributors to opioid toxicity harms that also captures non-fatal toxicities. While available national and provincial data have estimated the relative contribution of prescribed and non-prescribed opioids to toxicity deaths [1,12], reliance on post-mortem toxicology records limits the ability to understand the role of prescribed opioids in non-fatal toxicity events, or to differentiate the role of prescribed opioids that were dispensed versus diverted to opioid-related harms. Therefore, linked hospital records and community pharmacy dispensing data can provide important insight into the relative contributions of prescribed versus non-prescribed opioids to toxicity events, information that is needed to help inform clinical practice, pain management, and opioid agonist treatment (OAT) provision. Our primary objective was to describe the prevalence of active exposure to prescribed opioids at the time of opioid toxicity hospitalizations and ED visits between 2018 and 2022 across 6 Canadian provinces, annually and stratified by patient characteristics and type of opioid dispensed.

## Methods

### Setting

We conducted a population-based repeated cross-sectional study of yearly opioid toxicities treated as an inpatient hospital admission in 6 Canadian provinces (British Columbia, Alberta, Saskatchewan, Manitoba, Ontario and Quebec) between January 1st, 2018 and December 31st, 2022. In a secondary analysis, we replicated our analysis among opioid toxicities treated in ED settings in the 5 provinces where these data were available (all except Manitoba). The study was approved by Unity Health Toronto’s Research Ethics Board (REB# 23–275). Additional approvals to facilitate data access in specific provinces included authorization by the University of Alberta Research Ethics Board (Pro00083807) for the use of Alberta data, and authorization for the use of Ontario data under section 45 of Ontario’s Personal Health Information Protection Act, which does not require review by a Research Ethics Board. The use of Québec data was authorized through a tripartite agreement between the Ministère de la santé et des services sociaux of Québec, the Régie de l’assurance maladie du Québec (RAMQ), and the Institut national d’excellence en santé et services sociaux (INESSS), which was approved by the Commission d’Accès à l’information du Québec and does not require review by a Research Ethics Board. Data across studied provinces were accessed between 18/01/2024 and 16/10/2024. This study is reported in accordance with the Strengthening the Reporting of Observational Studies in Epidemiology (STROBE) guidelines [13] (**S1 File**).

### Data sources

We identified opioid prescriptions dispensed from community pharmacies over the study period using dispensing data from each included province. To capture pharmacy dispensing data across these provinces, we used four sources: (1) Canadian Institute for Health Information (CIHI) National Prescription Drug Utilization Information System for British Columbia, Saskatchewan and Manitoba; (2) the Narcotics Monitoring System for Ontario; (3) the Alberta Pharmaceutical Information Network for Alberta; and (4) the RAMQ database for Quebec. Each database contains information on all claims for pharmaceutical opioids dispensed from community pharmacies, regardless of payer in the province, except for RAMQ which only captures claims for people insured by Quebec’s public drug insurance plan. Therefore, coverage in Quebec is limited to people aged 65 and older, on social assistance, or without a private insurance plan which represents just under half of Quebec’s population [14].

We identified acute inpatient hospitalizations in British Columbia, Alberta, Saskatchewan, Manitoba, and Ontario using the CIHI Discharge Abstract Database whereas in Quebec we used the comparable database, Maintenance et exploitation des données pour l’étude de la clientèle hospitalière. To capture ED visits in British Columbia, Alberta, Saskatchewan and Ontario, we used the National Ambulatory Care Reporting System (NACRS), and for Quebec we used the Banque de données communes des urgences. Manitoba was not included in ED analyses as submission of data required to identify opioid toxicity visits to NACRS was not mandated over the study period. Similarly, we restricted the ED analyses in Saskatchewan to April 2021 onwards, to capture data following mandatory submission of full ICD-10-CA diagnosis codes to NACRS. In Saskatchewan and British Columbia, not all ED facilities submitted to NACRS over the study period and therefore ED data captured is incomplete in these provinces. In Ontario, all datasets were linked using anonymized unique encoded identifiers and analyzed at ICES. Data from British Columbia, Saskatchewan, and Manitoba were linked and analyzed at CIHI, data from Alberta were linked by Alberta Health Services and analyzed by the Alberta Drug and Technology and Technology Evaluation Consortium, and data from Quebec were linked and analyzed by the INESSS. All analyzed data across provinces were anonymized.

### Cohort definition

We identified all episodes of opioid toxicity (i) hospital admissions or (ii) ED visits separately in each province where data was available using a common protocol [15]. Opioid toxicity was defined using the International Classification of Diseases, 10^th^ revision (ICD-10) diagnosis codes T40.0, T40.1, T40.2, T40.3, T40.4, or T40.6. We flagged the intention of opioid toxicity using ICD-10 external cause of injury codes (E-codes): accidental (X42), intentional (X62), and unknown (all others).

We included all inpatient hospital stays where opioid toxicity diagnoses were identified, unless the toxicity was determined to have occurred post-admission (i.e., toxicities occurring during hospital admission), and excluded repeated admissions from the same episode of care. The one exception to this was Quebec, where the data source did not allow us to exclude post-admission diagnoses or repeat admissions (see **S1 Table** **for full details across province**s)

We captured all ED visits with opioid toxicity diagnoses. We excluded repeated ED visits within the same episode of care (e.g., transfers across multiple EDs were counted as one visit) across all provinces with the exception of Quebec and Alberta where this was not possible and so each ED record was considered to be an individual episode of care.

We defined the index date as the date of hospital admission or ED registration date. We excluded patients who had suspected diagnoses, missing/invalid patient identifiers, missing/invalid age or sex, were older than 105 at index, and who were residing outside of the province of interest on index date. If a patient had multiple opioid toxicity hospitalizations or ED visits over the study period, all events were included in each cohort. We considered the hospitalization and ED cohorts separately in our analyses, meaning that ED visits that led to a hospitalization were captured in both cohorts. In Quebec, we restricted the analysis to people who were continuously covered by the public drug insurance plan beginning in the 180 days prior to the first day of the year of interest until the end of the year, or date of death if applicable.

### Exposure definition

Our primary exposure variable was an active opioid exposure at the time of an opioid toxicity event defined as those with: (1) opioid prescriptions dispensed in the 100 days before (not including) the index date (i.e., the date of hospital admission) with a duration (days’ supply) overlapping the index date or (2) any methadone or oral buprenorphine product dispensed on the day prior to index date. The latter criterion was included to account for the commonly observed practice of daily dispensing of OAT products. We also examined recent opioid exposure, defined as any opioid prescriptions dispensed in the (i) 30 days and (ii) 180 days prior to, but not including, the index date. We captured dispensing of opioids indicated for pain, OAT (i.e., methadone, buprenorphine-naloxone, subcutaneous or implantable buprenorphine), and slow-release oral morphine (SROM) (i.e., Kadian®)―reported separately due to its mixed indication for both pain and OAT. We did not include claims for opioids used as cough suppressants or antidiarrheal (see **S2 Table** for list of opioid drug classes included in our study).

### Statistical analyses

We reported the number of opioid toxicity hospitalizations in each year over the study period. We described patient characteristics (including age, sex, and intention of toxicity) of all episodes of opioid toxicity hospitalizations in 2022, using descriptive statistics. We reported numbers and proportions of opioid toxicity hospitalizations with active opioid exposure annually over the study period (2018–2022), and stratified by age and sex for the year 2022 only. We described opioid toxicity hospitalizations with active opioid exposure by specific type of opioid dispensed (i.e., opioids for pain (any and by type), any OAT, any SROM) in the calendar years 2018 and 2022. We also reported the number and proportion of opioid toxicity hospitalizations with opioid exposure in the prior 30 days and 180 days, in each year over the study period. In a secondary analysis, we replicated all aforementioned analyses among opioid toxicity ED visits. All measures were reported separately in each province.

## Results

Across the 6 Canadian provinces, we identified 23,876 opioid toxicity hospitalizations over the study period, rising from 4,557 in 2018 to 4,841 in 2022 (see **S3 Table** for breakdown of numbers and rates in each province by year). Among hospital admissions for opioid toxicities in 2022, 1,632 occurred in British Columbia, 803 occurred in Alberta, 205 occurred in Saskatchewan, 106 occurred in Manitoba, 1,846 occurred in Ontario, and 249 occurred in Quebec (Table 1). Further, more than half of opioid toxicity hospitalizations occurred among people aged 25–64 across all provinces, ranging from 55.8% in Quebec to 77.8% in British Columbia. People hospitalized in Quebec tended to be older, with proportionally more opioid toxicity hospitalizations in both the 65–74 and 75 + age groups (35.0%) compared to other provinces (range 11.1%−20.8%). Over half of opioid toxicity hospitalizations occurred among males in British Columbia, Alberta, and Ontario (66.1%, 56.5%, and 58.5%, respectively), whereas proportions were similar across sexes in Saskatchewan, Manitoba, and Quebec (49.8%, 50.9%, 51.0% males, respectively). In provinces where reportable, most opioid toxicities treated in-hospital were accidental (range, 64.2%−75.3%) (**Table 1**).

**Table 1:** Descriptive characteristics of opioid toxicity hospital admissions in 2022.

	British Columbia N = 1632	Alberta N = 803	Saskatchewan N = 205	Manitoba N = 106	Ontario N = 1846	Quebec N = 249
**Age category (N, %)**						
0-24	182 (11.2%)	105 (13.1%)	30 (14.6%)	14 (13.2%)	169 (9.2%)	23 (9.2%)
25-44	698 (42.8%)	335 (41.7%)	93 (45.4%)	37 (34.9%)	688 (37.3%)	58 (23.3%)
45-64	571 (35.0%)	248 (30.9%)	59 (28.8%)	33 (31.1%)	605 (32.8%)	81 (32.5%)
65-74	122 (7.5%)	75 (9.3%)	11 (5.4%)	15 (14.2%)	241 (13.1%)	48 (19.3%)
≥75	59 (3.6%)	40 (5.0%)	12 (5.9%)	7 (6.6%)	143 (7.7%)	39 (15.7%)
**Sex (N, %)**						
Male	1078 (66.1%)	454 (56.5%)	102 (49.8%)	54 (50.9%)	1079 (58.5%)	127 (51.0%)
Female	554 (33.9%)	348 (43.3%)	103 (50.2%)	52 (49.1%)	767 (41.5%)	122 (49.0%)
**Intention of toxicity (N, %)**						
Accidental	1209 (74.1%)	605 (75.3%)	154 (75.1%)	68 (64.2%)	1192 (64.6%)	n/a
Intentional	282 (17.3%)	150 (18.7%)	32 (15.6%)	24 (22.6%)	438 (23.7%)	n/a
Unknown	141 (8.6%)	48 (6.0%)	19 (9.3%)	14 (13.2%)	216 (11.7%)	n/a

n/a = not available.

Note: Data on intention of toxicity was not available in Quebec.

Active opioid exposure at the time of opioid toxicity hospital admission varied across provinces, with the lowest proportion observed in British Columbia (range, 18.0% − 20.0%) and the highest proportion in Quebec (range, 36.5% − 48.2%) in each year over the study period (Table 2). In general, the proportion of opioid toxicity hospitalizations with active opioid exposure decreased between 2018 and 2022 with largest declines generally occurring in 2021 and 2022. The largest relative declines in active opioid exposure occurred in Manitoba (from 44.9% to 27.4%), Quebec (from 47.5% to 36.5%), and Alberta (from 34.9% to 27.5%). In contrast, British Columbia was the only province where the proportion of hospitalizations with active opioid exposure remained relatively stable over the study period (20.0% in 2018 vs. 19.7% in 2022). By 2022, the proportion of opioid toxicity hospitalizations with active opioid exposure was highest in Quebec (36.5%), followed by Saskatchewan (34.1%), Ontario (33.9%), Alberta (27.5%), Manitoba (27.4%) and British Columbia (19.7%) (Table 2). In our analyses considering any opioid dispensations in the prior 30 days, the proportion of hospitalizations with opioid exposure was higher across all provinces, ranging from 33.3% (British Columbia) to 50.6% (Quebec) in 2022. Proportions were higher when this criteria was lengthened to 180 days, ranging from 45.6% (British Columbia) to 60.2% (Quebec) in 2022 (**S6 Table**).

**Table 2:** Proportion of opioid toxicity hospitalizations and ED visits with active opioid exposure, 2018 to 2022.

	2018	2019	2020	2021	2022
**Hospital admissions**					
British Columbia	246 (20.0%)	187 (18.0%)	275 (19.7%)	331 (19.5%)	321 (19.7%)
Alberta	296 (34.9%)	261 (40.2%)	282 (34.3%)	298 (28.2%)	221 (27.5%)
Saskatchewan	78 (38.4%)	82 (35.3%)	94 (33.7%)	91 (32.9%)	70 (34.1%)
Manitoba	40 (44.9%)	28 (38.9%)	42 (40.8%)	21 (19.6%)	29 (27.4%)
Ontario	755 (39.1%)	692 (38.0%)	655 (34.5%)	738 (33.0%)	625 (33.9%)
Quebec	123 (47.5%)	118 (43.7%)	136 (48.2%)	102 (42.1%)	91 (36.5%)
**Emergency department visits**					
British Columbia	358 (11.0%)	436 (13.6%)	615 (15.2%)	968 (16.3%)	758 (16.8%)
Alberta	881 (17.1%)	744 (20.0%)	760 (16.5%)	1075 (14.7%)	816 (14.9%)
Saskatchewan	n/a	n/a	n/a	232 (19.0%)	244 (17.6%)
Ontario	2110 (24.4%)	2189 (22.4%)	2617 (22.5%)	3640 (23.5%)	2185 (20.3%)
Quebec	189 (28.5%)	176 (24.7%)	188 (24.3%)	225 (29.3%)	177 (26.0%)

n/a = not available. Note: For ED analyses, data is only available from April 2021 onwards in Saskatchewan. Denominators (i.e., number of opioid toxicities) in each year across provinces are presented in S3 Table and S4 Table, for the hospitalization and ED analyses, respectively.

When stratified by age, active opioid exposure at the time of hospitalization was highest among people aged 65 and older across all studied provinces (Table 3). For example, in 2022, over 55% of those aged 65 − 74 years had an active opioid exposure across all provinces (range, 58.3%−81.8%) apart from British Columbia (39.3%), and more than half of people 75 and older had an active opioid exposure across all provinces. Females were proportionally more likely to have an active opioid exposure than males in British Columbia (23.5% vs 17.7%), Alberta (34.2% vs 22.5%), Manitoba (38.5% vs 16.7%), Ontario (40.0% vs 29.5%), and Quebec (41.8% vs 31.5%); however, in Saskatchewan, proportions were similar between sexes (34.0% vs 34.3%) (**Table 3**).

**Table 3:** Proportion of opioid toxicity hospitalizations with active opioid exposure in 2022, stratified by age and sex.

	British Columbia	Alberta	Saskatchewan	Manitoba	Ontario	Quebec
**Age category (N, %)**						
0-24	13 (7.1%)	1-9 (0.1%−8.6%)	0 (0.0%)	0 (0.0%)	1-5 (0.6%−3.0%)	1-4 (4.4%−17.4%)
25-44	75 (10.7%)	56 (16.7%)	22 (23.7%)	1-4 (2.7%−10.8%)	126 (18.3%)	7-10 (12.1%−17.2%)
45-64	149 (26.1%)	88 (35.5%)	31 (52.5%)	10 (30.3%)	261−265 (43.1%−43.8%)	32 (39.5%)
65-74	48 (39.3%)	48 (64.0%)	9 (81.8%)	10 (66.7%)	147 (61.0%)	28 (58.3%)
≥75	36 (61.0%)	20-28 (50.0%−70.0%)	8 (66.7%)	4-7 (57.1%−100.0%)	86 (60.1%)	20 (51.3%)
**Sex (N, %)**						
Male	191 (17.7%)	102 (22.5%)	35 (34.3%)	9 (16.7%)	318 (29.5%)	40 (31.5%)
Female	130 (23.5%)	119 (34.2%)	35 (34.0%)	20 (38.5%)	307 (40.0%)	51 (41.8%)

Note: In accordance with privacy policies, non-zero small cell counts have been censored (N < 5 in British Columbia, Saskatchewan, Manitoba, and Quebec, N < 6 in Ontario, and N < 10 in Alberta). In cases where only one number is censored and the total number is provided, the next smallest cell for the stratification was suppressed to prevent residual disclosure. Denominators (i.e., number of opioid toxicities within each strata) across provinces are presented in Table 1.

Among opioid toxicity hospitalizations, exposure to different opioid types at time of admission changed between 2018 and 2022, with active exposure to opioids for pain becoming less common over time across all provinces. The largest absolute changes between 2018 and 2022 occurred in Manitoba (from 42.7% to 27.4%) and Quebec (from 45.2% to 32.9%), whereas the smallest changes occurred in British Columbia (14.9% to 10.2%), Saskatchewan (27.1% to 22.0%), and Ontario (30.5% to 25.5%; Table 4; S9 **Table**). In contrast, active exposure to OAT at time of hospitalization increased over time in Alberta (2.1% to 5.9%), British Columbia (5.3% to 9.6%), Quebec (N < 5 to 4.0%), and Saskatchewan (10.3% to 12.7%) and remained relatively unchanged in Ontario (9.1% to 8.7%). In Manitoba, active OAT exposure at time of hospitalization was exceedingly rare. Similarly, active exposure to SROM at the time of toxicity was low across all provinces in both 2018 and 2022 (**Table 4**; **S9 Table**).

**Table 4:** Proportion of opioid toxicity hospitalizations and ED visits with active opioid exposure in 2022, by type of opioid dispensed.

	British Columbia	Alberta	Saskatchewan	Manitoba	Ontario	Quebec
**Hospital admissions**						
**Any opioid for pain**	167 (10.2%)	174 (21.7%)	45 (22.0%)	29 (27.4%)	470 (25.5%)	82 (32.9%)
Oxycodone	24 (1.5%)	44 (5.5%)	N < 5	11 (10.4%)	159 (8.6%)	10 (4.0%)
Morphine	23 (1.4%)	23 (2.9%)	N < 5	N < 5	82 (4.4%)	12 (4.8%)
Codeine	44 (2.7%)	51 (6.4%)	6 (2.9%)	8 (7.5%)	63 (3.4%)	N < 5
Hydromorphone	72 (4.4%)	53 (6.6%)	28 (13.7%)	8 (7.5%)	194 (10.5%)	53 (21.3%)
Fentanyl	13 (0.8%)	16 (2.0%)	6 (2.9%)	6 (5.7%)	24 (1.3%)	10 (4.0%)
Other	14 (0.9%)	14 (1.7%)	6 (2.9%)	N < 5	28 (1.5%)	N < 5
**Any OAT**	156 (9.6%)	47 (5.9%)	26 (12.7%)	0 (0.0%)	161 (8.7%)	10 (4.0%)
**SROM**	9 (0.6%)	N < 10	N < 5	0 (0.0%)	8 (0.4%)	N < 5
**Emergency department visits**						
**Any opioid for pain**	122 (2.7%)	507 (9.2%)	110 (7.9%)	n/a	915 (8.5%)	104 (15.3%)
Oxycodone	21 (0.5%)	119 (2.2%)	8 (0.6%)	n/a	342 (3.2%)	17 (2.5%)
Morphine	8 (0.2%)	39 (0.7%)	8 (0.6%)	n/a	119 (1.1%)	19 (2.8%)
Codeine	28 (0.6%)	222 (4.0%)	18 (1.3%)	n/a	123 (1.1%)	N < 5
Hydromorphone	46 (1.0%)	99 (1.8%)	76 (5.5%)	n/a	356 (3.3%)	58 (8.5%)
Fentanyl	18 (0.4%)	18 (0.3%)	6 (0.4%)	n/a	38 (0.4%)	16 (2.3%)
Other	9 (0.2%)	49 (0.9%)	N < 5	n/a	44 (0.4%)	N < 5
**Any OAT**	651 (14.4%)	316 (5.8%)	134 (9.7%)	n/a	1290 (12.0%)	74 (10.9%)
**SROM**	7 (0.2%)	N < 10	14 (1.0%)	n/a	41 (0.4%)	N < 5

OAT= opioid agonist treatment; SROM = slow-release oral morphine; n/a = not available.

Note: In accordance with privacy policies, non-zero small cell counts have been censored (N < 5 in British Columbia, Saskatchewan, Manitoba, and Quebec, N < 6 in Ontario, and N < 10 in Alberta). Denominators (i.e., total number of opioid toxicities in 2022) across provinces are presented in S3 Table and S4 Table for the hospitalization and ED analyses, respectively. Multiple opioid types may be reported for one opioid toxicity hospitalization or ED visit when an individual is being actively dispensed more than one opioid type; therefore, categories are not mutually exclusive.

### Secondary analysis: opioid toxicities treated in an ED

In the secondary analysis of opioid toxicities treated in EDs, we identified 109,772 events across all provincial datasets over the study period (see **S4 Table** for breakdown of numbers and rates in each province per year). In 2022, the number of opioid toxicity ED visits were highest in Ontario (N = 10,772), followed by Alberta (N = 5,493), British Columbia (N = 4,519), Saskatchewan (N = 1,386), and Quebec (N = 681). Demographic characteristics were similar across provinces. Compared to those hospitalized, people with toxicities treated in the ED were proportionally more likely to be males and in the 25–44 age group across all provinces (**S5 Table**).

Active opioid exposure at the time of opioid toxicity ED visit decreased over time across most provinces where data was available throughout the study period, although to a lesser extent than that observed among toxicities that required a hospitalization (**Table 2**). Specifically, proportions declined in Alberta (from 17.1% to 14.9%), Ontario (from 24.4% to 20.3%), and Quebec (from 28.5% to 26.0%). The exception was British Columbia where the proportions of ED visits with active opioid exposure increased over time from 11.0% in 2018 to 16.8% in 2022. In 2022, active opioid exposure at the time of ED visit was lower than that observed at the time of hospital admission in all provinces. Specifically, the proportion was highest in Quebec (26.0%), followed by Ontario (20.3%), Saskatchewan (17.6%), British Columbia (16.8%), and Alberta (14.9%) (**Table 2**). Similar to our primary analysis, the proportions of opioid toxicity visits with opioid exposure increased when alternative definitions were used limiting prior dispensations to 30 and 180 days (**S7 Table**). Patterns of active opioid exposure across demographic characteristics were generally consistent between opioid toxicity ED visits and hospitalizations, with higher proportions of ED visits with an active opioid exposure among females and people aged 65 and older across most provinces. Notably, in British Columbia, active opioid exposure was slightly lower among people aged 65–74 (29.6%) and ≥75 (34.2%), compared to the other provinces (range 50.0%–71.0% and 48.5%−75.0%, respectively; **S8 Table**).

Active exposure to opioids for pain was much lower among opioid toxicities treated in the ED (range, 2.7% to 15.3% in 2022) compared to those that required a hospital admission (range, 10.2%−32.9% in 2022), although proportions were similarly lowest in British Columbia and highest in Quebec (**Table 4**). Moreover, in 2022, active exposure to OAT was more common than exposure to opioids indicated for pain in British Columbia (14.4% vs 2.7%), Saskatchewan (9.7% vs 7.9%), and Ontario (12.0% vs 8.5%), whereas in Alberta and Quebec exposure to opioids for pain was higher (5.8% vs 9.2% and 10.9% vs 15.3%, respectively). Among opioid toxicity ED visits, active exposure to opioids for pain slightly declined over time (range, 3.2%–22.9% in 2018 vs 2.7%–15.3% in 2022), while active exposure to OAT slightly increased across provinces (range of 2.6%–10.4% in 2018 vs. 5.8%%–14.4% in 2022). Active exposure to SROM at the time of ED visit was low in both 2018 and 2022 (**Table 4**; **S9 Table**).

## Discussion

In this population-based study, we examined exposure to prescribed opioids prior to opioid toxicity hospitalizations and ED visits between 2018 and 2022, across 6 Canadian provinces. We found that active opioid exposure before hospital-treated toxicities declined over time across Canada, and that toxicities treated in EDs were less likely to occur among people with an active opioid exposure. Overall, by 2022, opioid toxicities treated in inpatient settings involved individuals actively treated with a prescription opioid in roughly one-fifth to one-third of cases (20%–37%), with lower proportions in emergency departments (15%–26%) across all provinces studied. Importantly, we observed notable differences in active opioid exposure at time of toxicity across geography and demographic groups, with people older than 65 and females being proportionally more likely to be receiving a prescription opioid at time of hospital visit, and those residing in British Columbia being less likely to be receiving a prescription opioid―particularly opioids indicated for pain. Generally, declining proportions of opioid toxicity visits with active opioid exposure was driven by decreased dispenses of opioids for pain, with notable increases in active OAT exposure observed over the study period. Taken together, our findings reinforce the growing dominance of non-pharmaceutical opioids from the unregulated drug supply as contributors to opioid-related harms across Canada, particularly among men and younger populations. This aligns with previous research up to 2016 that signaled rising exposure through non-pharmaceutical opioids among people presenting to acute-care settings with an opioid toxicity diagnosis in some Canadian jurisdictions, as fentanyl emerged in the unregulated drug supply [12,16]. Our study shows how patterns have shifted since 2016, in line with the evolution of the opioid landscape across different regions in the country. Given the observed differences across jurisdictions and demographic groups reported in our study, it is imperative that policies designed to address the opioid toxicity crisis consider the nuanced and changing role of pharmaceutical and non-pharmaceutical opioid sources as drivers of harms in Canada.

Despite the overall low proportion of active opioid exposure relative to opioid toxicity events across provinces, there were notable inter-provincial variations. For example, in 2022, the proportion of hospitalizations with active opioid exposure in British Columbia (19.7%) was almost two-fold lower than in Quebec (36.5%). This likely reflects geographical differences in opioid prescribing practices, the potency of the unregulated drug supply, and differences in populations captured in the underlying data in our study. First, fentanyl has been the dominant opioid in British Columbia’s unregulated drug supply since at least 2016 [5,17], and was detected in over 80% of unregulated opioid samples tested in British Columbia in 2022 [5]. In comparison, in the same year, the proportion of fentanyl in unregulated opioid samples tested in Quebec was 19.1%―more than three-fold lower than observed in other included provinces [5]. Given the established role that fentanyl has had in driving rising toxicity rates throughout Canada and the U.S., differences in the potency of the unregulated drug supply are likely influencing the patterns observed in this study. However, our findings also likely reflect the restricted population captured in Quebec data, where only those eligible for the public drug program are included (in contrast to full population data available in all other included provinces). Therefore, the Quebec population is likely more concentrated in an older demographic that is more likely to experience toxicities involving prescription opioids [1]. Importantly, despite the higher prevalence of active exposure to prescribed opioids in Quebec generally, this has decreased between 2018 and 2022, aligning with reports of increased unregulated fentanyl involvement in opioid-related harms in this province in recent years [1]. Similarly, we observed declining active opioid exposure among opioid toxicity hospitalizations in Alberta and Manitoba (39.0% and 21.2% decrease, respectively), two provinces that have seen rapid accelerations in opioid-related deaths involving fentanyl since 2019 and notable growths in fentanyl detection in the unregulated drug supply over a similar period [1,3,5]. Therefore, our findings not only reinforce the important and growing role that the unregulated supply is having on opioid toxicity rates broadly across the country, but also mirrors regional differences in the drivers of opioid-related harms and how these have evolved over time.

In general, toxicities among women and older age groups were more likely to involve exposure to prescription opioids, a pattern which mirrors broader patterns of opioid analgesic prescribing [6,18–21], and is generally attributed to higher rates of pain in these populations [22]. National data on opioid toxicity harms have also demonstrated that fatal toxicities from the unregulated drug supply are more concentrated among younger men [1]. Taken together, these contextual factors may explain our findings given existing broader patterns of demographic differences in opioid exposure and related harms across age and sex. Importantly, potential variations in the role of prescription opioids and the unregulated drug supply across demographic groups suggests the need for tailored substance-related programs and services that take into account the unique needs of different populations. For example, dual strategies that target safe prescribing and pharmaceutical opioid use among older populations, and harm reduction services and evidence-based treatment for opioid use disorder (OUD) in younger populations should be prioritized.

Finally, evidence of active exposure to OAT before opioid toxicity increased over time in most provinces. This may be reflective of a growing population of people who require treatment for OUD throughout Canada in the context of the fentanyl-dominated drug supply and national efforts made to remove barriers to treatment for people with OUD [6,23–25]. However, we may also be capturing toxicities among individuals who abruptly discontinued OAT, given that overdose risks increase considerably during this period. While strategies such as the expansion of virtual treatment models and extended-release longer acting forms of treatment are designed to increase access to OAT, evidence suggests that rates of OAT retention are low across Canada and have been declining as the potency of the unregulated drug supply has increased [26,27]. Therefore, there is a need to consider how OAT programs can be adapted to help minimize risks of toxicity early in treatment, support longer-term retention, as well as planned tapering when desired by patient to help mitigate harms among people engaging in OAT.

The primary strengths of our study includes the use of linked population-based databases to characterize patterns of exposure to prescribed opioids among opioid toxicity hospitalizations and ED visits across the majority of Canada’s population. However, there are several notable limitations that warrant discussion. First, we were unable to determine whether prescribed opioids were directly involved in opioid toxicity events, or whether toxicities occurred as a result of the unregulated drug supply. Therefore, while this study provides helpful insight into the changing role of prescribed opioids in opioid-related harms across Canada, they may underestimate the potential role of diverted prescription opioids and the unregulated drug supply. Second, our definition of active exposure to OAT only captures methadone and buprenorphine (alone or in combination with naloxone), excluding SROM 24-hour formulation, as we could not differentiate use to treat pain versus OUD. However, our analyses point to a very small percentage of people with active exposure to SROM across provinces. Additionally, we were unable to differentiate immediate release hydromorphone dispensing for pain vs safer opioid supply, and thus, we categorized all dispensing of this opioid as indicated for pain in our analyses. However, given small exposure rates to safer opioid supply across the country, we expect the influence on trends to be minimal. Third, in Quebec, only prescription drug claims made to the public drug program were captured, meaning that results from this jurisdiction are not population-based, and patterns observed may differ from those without public drug insurance coverage. Moreover, in Quebec, we could not exclude toxicities occurring while admitted (although we anticipate this number to be small based on exclusions in other provinces). Fourth, British Columbia and Saskatchewan also had partial ED data, as not all ED facilities submitted data to NACRS over the study period, however coverage of all ED visits in these provinces is estimated to be over 70% and 80%, respectively, in the study period captured [28]. Therefore, analyses in these jurisdictions may not be representative of the entirety of the province and most importantly, absolute numbers of opioid toxicity ED visits in these provinces are an underestimate. Fifth, we only captured opioid-toxicity events that presented to the hospital or ED and not toxicities that presented in other community-based settings. Finally, although, there are documented socio-economic differences across our studied provinces [4], we were unable to account for this in our analysis.

## Conclusions

Overall, this study further reinforces the greatly reduced role of prescribed opioids as a driver of opioid-related harms across Canada. Specifically, more than half of opioid toxicity hospitalizations and ED visits did not have any evidence of a dispensed prescription opioid, and this prevalence decreased over time. Further, among toxicities where people were actively exposed to prescribed opioids, OAT was increasingly identified as the opioid dispensed, pointing to the declining contribution of prescribed opioid analgesics to harms across the country. However, our findings also reveal variability in exposure to prescribed opioids across jurisdictions and demographic groups. Therefore, as the substance-related toxicity crisis persists across the country, our findings suggest a need for policy responses that target the various drivers of drug-related harms across Canada, with a particular focus on programs and services designed to reduce harms from the fentanyl-dominated unregulated drug supply.

## Supporting information

S1 File STROBE checklist. (DOCX)

S1 Table Diagnoses types used to determine opioid-toxicity event present at hospital admission. (DOCX)

S2 Table Opioid types and drug classes captured in analyses. (DOCX)

S3 Table Number of opioid toxicity hospital admissions, 2018–2022. (DOCX)

S4 Table Number of opioid toxicity ED visits, 2018–2022. (DOCX)

S5 Table Descriptive characteristics of opioid toxicities treated in the ED in 2022. (DOCX)

S6 Table Proportion of opioid toxicity hospitalizations with opioid exposure in the prior 30 and 180 days, 2018–2022. (DOCX)

S7 Table Proportion of opioid toxicity ED visits with opioid exposure in the prior 30 and 180 days, 2018–2022. (DOCX)

S8 Table Proportion of opioid toxicity ED visits with active opioid exposure in 2022, stratified by age and sex. (DOCX)

S9 Table Proportion of opioid toxicity hospitalizations and ED visits with active opioid exposure in 2018, by type of opioid dispensed. (DOCX)
