# Supplementary material for: Opioid dispensing prior to opioid toxicity hospitalizations and emergency department visits in Canada, 2018–2022
Source: PLoS One. 2026 Jan 12;21(1):e0339643. doi: 10.1371/journal.pone.0339643 (PMC12795387; doi:10.1371/journal.pone.0339643)
Supplement: S1 Table — (DOCX) [file pone.0339643.s002.docx]

**SUPPORTING INFORMATION**

**S1 Table. Diagnoses types used to determine opioid-toxicity event present at hospital admission.**

| **Provinces** | **Admission Diagnoses Definition** |
| --- | --- |
| **British Columbia** | Records with at least one Diagnosis for Opioid-Related Toxicity (Appendix 1) with one of:   - Diagnosis Type M (most responsible diagnosis) - Diagnosis Type 1 (pre-admit comorbidity) - Diagnosis Type W, X, Y (service transfer diagnosis)   Exclusions:   - Records that have a Diagnosis for Opioid-Related Toxicity as Diagnosis Type 2 (post-admit comorbidity) |
| **Alberta** | Records with at least one Diagnosis for Opioid-Related Toxicity (Appendix 1) with one of:   - Diagnosis Type 1 (pre-admit comorbidity) - Diagnosis Type W, X, Y (service transfer diagnosis) - Diagnosis Type M (most responsible diagnosis) as long as Diagnosis for Opioid-Related Toxicity does not show up with Diagnosis Type 2 (post-admit comorbidity) on the same record - Diagnosis Type 5 (admitting diagnosis) (optional) |
| **Saskatchewan** | Records with at least one Diagnosis for Opioid-Related Toxicity (Appendix 1) with one of :   - Diagnosis Type M (most responsible diagnosis) - Diagnosis Type 1 (pre-admit comorbidity) - Diagnosis Type W, X, Y (service transfer diagnosis)     Exclusions:   - Records that have a Diagnosis for Opioid-Related Toxicity as Diagnosis Type 2 (post-admit comorbidity) |
| **Manitoba** | Records with at least one Diagnosis for Opioid-Related Toxicity (Appendix 1) with one of:   - Diagnosis Type M (most responsible diagnosis) - Diagnosis Type 1 (pre-admit comorbidity) - Diagnosis Type W, X, Y (service transfer diagnosis)     Exclusions:   - Records that have a Diagnosis for Opioid-Related Toxicity as Diagnosis Type 2 (post-admit comorbidity) |
| **Ontario** | Records with at least one Diagnosis for Opioid-Related Toxicity (Appendix 1) with one of:   - Diagnosis Type 1 (pre-admit comorbidity) - Diagnosis Type W, X, Y (service transfer diagnosis) - Diagnosis Type M (most responsible diagnosis) as long as Diagnosis for Opioid-Related Toxicity does not show up with Diagnosis Type 2 (post-admit comorbidity) on the same record |
| **Quebec** | Records with at least one Diagnosis for Opioid-Related Toxicity (Appendix 1) with one of:   - Diagnosis Type M (with Diagnosis Type 2 [post-admit comorbidity] included) - Diagnosis Type 5 (admitting diagnosis) (optional) |
