# Supplementary material for: Opioid dispensing prior to opioid toxicity hospitalizations and emergency department visits in Canada, 2018–2022
Source: PLoS One. 2026 Jan 12;21(1):e0339643. doi: 10.1371/journal.pone.0339643 (PMC12795387; doi:10.1371/journal.pone.0339643)
Supplement: S2 Table — (DOCX) [file pone.0339643.s003.docx]

**S2 Table. Opioid types and drug classes captured in analyses.**

| **Opioid type** | **Drug Class** |
| --- | --- |
| **Opioids for pain** | Oxycodone  Morphine  Codeine  Hydromorphone  Fentanyl  Other (buprenorphine [pain]), butorphanol, dextropropoxyphene, meperidine, methadone [pain], nalbuphine, oxymorphone, pentazocine, tapentadol, or tramadol) |
| **Opioid Agonist Therapy** | Methadone  Buprenorphine (buprenorphine/naloxone, subcutaneous buprenorphine extended-release [Sublocade] or implantable buprenorphine [Probuphine) |
| **Slow-Release Oral Morphine** | Slow-release oral morphine [Kadian 24-hour formulation] |
