# Supplementary material for: Opioid dispensing prior to opioid toxicity hospitalizations and emergency department visits in Canada, 2018–2022
Source: PLoS One. 2026 Jan 12;21(1):e0339643. doi: 10.1371/journal.pone.0339643 (PMC12795387; doi:10.1371/journal.pone.0339643)
Supplement: S3 Table — (DOCX) [file pone.0339643.s004.docx]

|  | **2018** | **2019** | **2020** | **2021** | **2022** |
| --- | --- | --- | --- | --- | --- |
|  | **N**  **(Rate per 1,000)** | **N**  **(Rate per 1,000)** | **N**  **(Rate per 1,000)** | **N**  **(Rate per 1,000)** | **N**  **(Rate per 1,000)** |
| **British Columbia** | 1,229 (0.24) | 1038 (0.20) | 1398 (0.27) | 1695 (0.32) | 1,632 (0.30) |
| **Alberta** | 847 (0.20) | 649 (0.15) | 822 (0.19) | 1057 (0.24) | 803 (0.18) |
| **Saskatchewan** | 203 (0.18) | 232 (0.20) | 279 (0.24) | 277 (0.24) | 205 (0.17) |
| **Manitoba** | 89 (0.07) | 72 (0.05) | 103 (0.07) | 107 (0.08) | 106 (0.07) |
| **Ontario** | 1,930 (0.13) | 1822 (0.13) | 1896 (0.13) | 2237 (0.15) | 1,846 (0.12) |
| **Quebec** | 259 (0.08) | 270 (0.08) | 282 (0.08) | 242 (0.07) | 249 (0.07) |
| **Total (N only)** | **4557** | **4083** | **4780** | **5615** | **4841** |

**S3 Table. Number and rate per 1,000 of opioid toxicity hospital admissions, 2018 to 2022.**

Note: Rates were calculated using provincial population estimates in each year from Statistics Canada, except for Quebec where we used population insured by the province’s public drug insurance plan.
