# Supplementary material for: Opioid dispensing prior to opioid toxicity hospitalizations and emergency department visits in Canada, 2018–2022
Source: PLoS One. 2026 Jan 12;21(1):e0339643. doi: 10.1371/journal.pone.0339643 (PMC12795387; doi:10.1371/journal.pone.0339643)
Supplement: S4 Table — (DOCX) [file pone.0339643.s005.docx]

**S4 Table. Number and rate per 1,000 of opioid toxicity ED visits, 2018 to 2022.**

|  | **2018** | **2019** | **2020** | **2021** | **2022** |
| --- | --- | --- | --- | --- | --- |
|  | **N**  **(Rate per 1,000)** | **N**  **(Rate per 1,000)** | **N**  **(Rate per 1,000)** | **N**  **(Rate per 1,000)** | **N**  **(Rate per 1,000)** |
| **British Columbia** | 3255 (n/a) | 3196 (n/a) | 4045 (n/a) | 5953 (n/a) | 4519 (n/a) |
| **Alberta** | 5167 (1.20) | 3716 (0.85) | 4614 (1.05) | 7292 (1.65) | 5493 (1.22) |
| **Saskatchewan** | n/a | n/a | n/a | 1224 (n/a) | 1386 (n/a) |
| **Ontario** | 8630 (0.60) | 9785 (0.67) | 11618 (0.79) | 15460 (1.04) | 10772 (0.71) |
| **Quebec** | 663 (0.20) | 713 (0.21) | 773 (0.22) | 767 (0.22) | 681 (0.19) |
| **Total (N only)** | **17,715** | **17,410** | **21,050** | **30,696** | **22,851** |

n/a = not available.

Note: Data is only available from April 2021 onwards for Saskatchewan. Data for Saskatchewan and British Columbia capture only a subset of all ED facilities in these provinces, therefore rates were not reportable due to partial data coverage. Where applicable, rates were calculated using provincial population estimates in each year from Statistics Canada, except for Quebec where we used population insured by the province’s public drug insurance plan.
