# Supplementary material for: Opioid dispensing prior to opioid toxicity hospitalizations and emergency department visits in Canada, 2018–2022
Source: PLoS One. 2026 Jan 12;21(1):e0339643. doi: 10.1371/journal.pone.0339643 (PMC12795387; doi:10.1371/journal.pone.0339643)
Supplement: S5 Table — (DOCX) [file pone.0339643.s006.docx]

**S5 Table. Descriptive characteristics of opioid toxicities treated in the ED in 2022.**

|  | **British Columbia**  **N=4519** | **Alberta**  **N=5493** | **Saskatchewan**  **N=1386** | **Ontario**  **N=10,772** | **Quebec**  **N=681** |
| --- | --- | --- | --- | --- | --- |
| **Age category (N, %)** |  |  |  |  |  |
| 0-24 | 421 (9.3%) | 891 (16.2%) | 249 (18.0%) | 1187 (11.0%) | 52 (7.6%) |
| 25-44 | 2453 (54.3%) | 3207 (58.4%) | 831 (60.0%) | 6265 (58.2%) | 322 (47.3%) |
| 45-64 | 1438 (31.8%) | 1193 (21.7%) | 263 (19.0%) | 2716 (25.2%) | 221 (32.5%) |
| 65-74 | 169 (3.7%) | 136 (2.5%) | 31 (2.2%) | 386 (3.6%) | 53 (7.8%) |
| ≥75 | 38 (0.8%) | 66 (1.2%) | 12 (0.9%) | 218 (2.0%) | 33 (4.8%) |
| **Sex (N, %)** |  |  |  |  |  |
| Male | 3319 (73.4%) | 3485 (63.4%) | 819 (59.1%) | 7344 (68.2%) | 450 (66.1%) |
| Female | 1200 (26.6%) | 2006 (36.5%) | 567 (40.9%) | 3428 (31.8%) | 231 (33.9%) |
| **Intention of toxicity (N, %)** |  |  |  |  |  |
| Accidental | n/a | 4703 (85.6%) | 1061 (76.6%) | 8421 (78.2%) | n/a |
| Intentional | n/a | 488 (8.9%) | 151 (10.9%) | 1127 (10.5%) | n/a |
| Unknown | n/a | 302 (5.5%) | 174 (12.6%) | 1224 (11.4%) | n/a |

n/a = not available.

Note: Data on intention of toxicity is not available in British Columbia and Quebec.
