# Supplementary material for: Opioid dispensing prior to opioid toxicity hospitalizations and emergency department visits in Canada, 2018–2022
Source: PLoS One. 2026 Jan 12;21(1):e0339643. doi: 10.1371/journal.pone.0339643 (PMC12795387; doi:10.1371/journal.pone.0339643)
Supplement: S6 Table — (DOCX) [file pone.0339643.s007.docx]

|  | **2018** | **2019** | **2020** | **2021** | **2022** |
| --- | --- | --- | --- | --- | --- |
| **Recent prescription opioid exposure: 30 days** | | |  |  |  |
| British Columbia | 363 (29.5%) | 321 (30.9%) | 444 (31.8%) | 582 (34.3%) | 544 (33.3%) |
| Alberta | 375 (44.3%) | 333 (51.3%) | 354 (43.1%) | 460 (43.5%) | 303 (37.7%) |
| Saskatchewan | 94 (46.3%) | 94 (40.5%) | 114 (40.9%) | 114 (41.2%) | 99 (48.3%) |
| Manitoba | 46(51.7%) | 35 (48.6%) | 48 (46.6%) | 28 (26.2%) | 38 (35.8%) |
| Ontario | 963 (49.9%) | 927 (50.9%) | 886 (46.7%) | 1091 (48.8%) | 867 (47.0%) |
| Quebec | 163 (62.9%) | 148 (54.8%) | 180 (63.8%) | 141 (58.3%) | 126 (50.6%) |
| **Recent prescription opioid exposure: 180 days** | | |  |  |  |
| British Columbia | 531 (43.2%) | 454 (43.7%) | 591 (42.3%) | 820 (48.4%) | 745 (45.6%) |
| Alberta | 496 (58.6%) | 417 (64.3%) | 458 (55.7%) | 615 (58.2%) | 422 (52.6%) |
| Saskatchewan | 114(56.2%) | 115 (49.6%) | 147 (52.7%) | 149 (53.8%) | 118 (57.6%) |
| Manitoba | 63 (70.8%) | 42 (58.3%) | 61 (59.2%) | 48 (44.9%) | 61 (57.5%) |
| Ontario | 1219 (63.2%) | 1165 (63.9%) | 1121 (59.1%) | 1360 (60.8%) | 1100 (59.6%) |
| Quebec | 174 (67.2%) | 174 (64.4%) | 203 (72.0%) | 170 (70.2%) | 150 (60.2%) |

**S6 Table. Proportion of opioid toxicity hospitalizations with opioid exposure in the prior 30 and 180 days, 2018 to 2022.**

Note: Denominators (i.e., number of opioid-related toxicities) in each year across provinces are presented in S3 Table.
