# Supplementary material for: Opioid dispensing prior to opioid toxicity hospitalizations and emergency department visits in Canada, 2018–2022
Source: PLoS One. 2026 Jan 12;21(1):e0339643. doi: 10.1371/journal.pone.0339643 (PMC12795387; doi:10.1371/journal.pone.0339643)
Supplement: S7 Table — (DOCX) [file pone.0339643.s008.docx]

**S7 Table. Proportion of opioid toxicity ED visits with opioid exposure in the prior 30 and 180 days, 2018 to 2022.**

|  | **2018** | **2019** | **2020** | **2021** | **2022** |
| --- | --- | --- | --- | --- | --- |
| **Recent prescription opioid exposure: 30 days** | | | | | |
| British Columbia | 757 (23.3%) | 1014 (31.7%) | 1400 (34.6%) | 2191 (36.8%) | 1618 (35.8%) |
| Alberta | 1390 (26.9%) | 1153 (31.0%) | 1317 (28.5%) | 2145 (29.4%) | 1499 (27.3%) |
| Saskatchewan | n/a | n/a | n/a | 350 (28.6%) | 369 (26.6%) |
| Ontario | 3108 (36.0%) | 3532 (36.1%) | 4296 (37.0%) | 6613 (42.8%) | 3613 (33.5%) |
| Quebec | 274 (41.3%) | 250 (35.1%) | 286 (37.0%) | 340 (44.3%) | 291 (42.7%) |
| **Recent prescription opioid exposure: 180 days** | | |  |  |  |
| British Columbia | 1259 (38.7%) | 1549 (48.5%) | 1967 (48.6%) | 3047 (51.2%) | 2210 (48.9%) |
| Alberta | 2215 (42.9%) | 1713 (46.1%) | 2009 (43.5%) | 3432 (47.1%) | 2409 (43.9%) |
| Saskatchewan | n/a | n/a | n/a | 530 (43.3%) | 548 (39.5%) |
| Ontario | 4493 (52.1%) | 5063 (51.7%) | 5936 (51.1%) | 8979 (58.1%) | 5117 (47.5%) |
| Quebec | 318 (48.0%) | 327 (45.9%) | 372 (48.1%) | 412 (53.7%) | 361 (53.0%) |

n/a = not available.

Note: Data is only available from April 2021 onwards for Saskatchewan. Denominators (i.e., number of opioid toxicities) in each year across provinces are presented in S4 Table.
