# Supplementary material for: Opioid dispensing prior to opioid toxicity hospitalizations and emergency department visits in Canada, 2018–2022
Source: PLoS One. 2026 Jan 12;21(1):e0339643. doi: 10.1371/journal.pone.0339643 (PMC12795387; doi:10.1371/journal.pone.0339643)
Supplement: S8 Table — (DOCX) [file pone.0339643.s009.docx]

|  | **British Columbia** | **Alberta** | **Saskatchewan** | **Ontario** | **Quebec** |
| --- | --- | --- | --- | --- | --- |
| **Age category (N, %)** |  |  |  |  |  |
| 0-24 | 32 (7.6%) | 31 (3.5%) | 5 (2.0%) | 59 (5.0%) | 1-4 (1.9%-7.7%) |
| 25-44 | 324 (13.2%) | 332 (10.4%) | 119 (14.3%) | 972 (15.5%) | 58-63 (18.0%-19.6%) |
| 45-64 | 339 (23.6%) | 342 (28.7%) | 89 (33.8%) | 831 (30.6%) | 73 (33.0%) |
| 65-74 | 50 (29.6%) | 73 (53.7%) | 22 (71.0%) | 193 (50.0%) | 29 (54.7%) |
| ≥75 | 13 (34.2%) | 38 (57.6%) | 9 (75.0%) | 130 (59.6%) | 16 (48.5%) |
| **Sex (N, %)** |  |  |  |  |  |
| Male | 587 (17.7%) | 451 (12.9%) | 135 (16.5%) | 1375 (18.7%) | 108 (24.0%) |
| Female | 171 (14.3%) | 365 (18.2%) | 109 (19.2%) | 810 (23.6%) | 69 (29.9%) |

**S8 Table. Proportion of opioid toxicity ED visits with active opioid exposure in 2022, stratified by age and sex.**

Note: In accordance with privacy policies, non-zero small cell counts have been censored (N<5 in British Columbia, Saskatchewan, Manitoba, and Quebec, N<6 in Ontario, and N<10 in Alberta). In cases where only one number is censored and the total number is provided, the next smallest cell for the stratification was suppressed to prevent residual disclosure. Denominators (i.e., number of opioid-related toxicities within each strata) across provinces are presented in S5 Table.
