# Supplementary material for: Opioid dispensing prior to opioid toxicity hospitalizations and emergency department visits in Canada, 2018–2022
Source: PLoS One. 2026 Jan 12;21(1):e0339643. doi: 10.1371/journal.pone.0339643 (PMC12795387; doi:10.1371/journal.pone.0339643)
Supplement: S9 Table — (DOCX) [file pone.0339643.s010.docx]

|  | **British Columbia** | **Alberta** | **Saskatchewan** | **Manitoba** | **Ontario** | **Quebec** |
| --- | --- | --- | --- | --- | --- | --- |
| **Hospital admissions** | | | | | | |
| **Any opioid for pain** | 183 (14.9%) | 278 (32.8%) | 55 (27.1%) | 38 (42.7%) | 589 (30.5%) | 117 (45.2%) |
| Oxycodone | 32 (2.6%) | 87 (10.3%) | N<5 | 8 (9.0%) | 206 (10.7%) | 15 (5.8%) |
| Morphine | 34 (2.8%) | 33 (3.9%) | N<5 | 7 (7.9%) | 78 (4.0%) | 25 (9.7%) |
| Codeine | 49 (4.0%) | 103 (12.2%) | 8 (3.9%) | 11 (12.4%) | 79 (4.1%) | N<5 |
| Hydromorphone | 58 (4.7%) | 55 (6.5%) | 37 (18.2%) | 13 (14.6%) | 247 (12.8%) | 57 (22.0%) |
| Fentanyl | 11 (0.9%) | 20 (2.4%) | 6 (3.0%) | N<5 | 39 (2.0%) | 27 (10.4%) |
| Other | 31 (2.5%) | 23 (2.7%) | 8 (3.9%) | N<5 | 32 (1.7%) | 11 (4.2%) |
| **Any OAT** | 65 (5.3%) | 18 (2.1%) | 21 (10.3%) | N<5 | 176 (9.1%) | N<5 |
| **SROM** | N<5 | N<10 | N<5 | 0 (0.0%) | 7 (0.4%) | N<5 |
| **Emergency department visits** | | | | | | |
| **Any opioid for pain** | 105 (3.2%) | 748 (14.5%) | n/a | n/a | 1238 (14.3%) | 152 (22.9%) |
| Oxycodone | 21 (0.6%) | 221 (4.3%) | n/a | n/a | 483 (5.6%) | 37 (5.6%) |
| Morphine | 10 (0.3%) | 58 (1.1%) | n/a | n/a | 163 (1.9%) | 29 (4.4%) |
| Codeine | 54 (1.7%) | 355 (6.9%) | n/a | n/a | 171 (2.0%) | 8 (1.2%) |
| Hydromorphone | 17 (0.5%) | 129 (2.5%) | n/a | n/a | 492 (5.7%) | 72 (10.9%) |
| Fentanyl | N<5 | 30 (0.6%) | n/a | n/a | 62 (0.7%) | 25 (3.8%) |
| Other | 7 (0.2%) | 45 (0.9%) | n/a | n/a | 50 (0.6%) | 10 (1.5%) |
| **Any OAT** | 254 (7.8%) | 133 (2.6%) | n/a | n/a | 899 (10.4%) | 33 (5.0%) |
| **SROM** | 5 (0.2%) | N<10 | n/a | n/a | 16 (0.2%) | 8 (1.2%) |

**S9 Table. Proportion of opioid toxicity hospitalizations and ED visits with active opioid exposure in 2018, by type of opioid dispensed.**

n/a = not available; OAT = opioid agonist treatment; SROM = slow-release oral morphine.

Note: In accordance with privacy policies, non-zero small cell counts have been censored (N<5 in British Columbia, Saskatchewan, Manitoba, and Quebec, N<6 in Ontario, and N<10 in Alberta). Denominators (i.e., total number of opioid-related toxicities in 2018) across provinces are presented in S3 Table and S4 Table for the hospitalization and ED analyses, respectively. ED data is only available from April 2021 onwards for Saskatchewan and Manitoba is not included in the ED analyses. Multiple opioid types may be reported for one opioid toxicity hospitalization or ED visit when an individual is being actively dispensed more than one opioid type; therefore, categories are not mutually exclusive.
